# Artificial Intelligence in the Detection and Risk Stratification of Choroidal Melanoma: A Critical Comparative Synthesis and Future Directions

**DOI:** 10.3390/healthcare13243252

**Published:** 2025-12-11

**Authors:** Daire Hurley, Amy Coman, Elizabeth Tallon, Noel Horgan, Patrick Murtagh

**Affiliations:** Department of Ophthalmology, Royal Victoria Eye and Ear Hospital, Adelaide Road, D02 XK51 Dublin, Ireland

**Keywords:** choroidal melanoma, naevus, artificial intelligence, AI

## Abstract

The early differentiation of benign choroidal naevi from malignant melanoma remains one of the most nuanced challenges in ophthalmic oncology, with profound implications for patient survival. Conventional diagnostic pathways rely on multimodal imaging and expert interpretation, but inter-observer variability and the rarity of melanoma limit timely and consistent detection. Recent advances in artificial intelligence (AI) offer a promising adjunct to conventional ophthalmic practice. This review provides a critical comparative synthesis of the studies to-date which have looked at AI’s use in the detection, risk stratification, and longitudinal monitoring of choroidal melanoma. While early results are promising—with some models achieving an accuracy comparable to expert clinicians—significant challenges remain regarding generalisability, dataset bias, interpretability, and real-world deployment. We conclude by outlining practical priorities for future research to ensure that AI becomes a safe, effective, and equitable tool for improving patient outcomes.

## 1. Introduction

Choroidal melanoma accounts for more than 80% of all uveal melanomas and is the most common primary intraocular cancer in adults in the U.S. population [1]. Although its annual incidence is relatively low—approximately five cases per million in Western countries—the impact is significant: metastatic disease develops in as many as 50% of patients by 10 years, primarily in the liver, despite local control of the primary tumour [2,3,4]. Once metastatic, the prognosis is poor, with a median survival of under a year [5]. By contrast, choroidal naevi are common benign pigmented lesions, present in up to 10% of the general population [6]. The vast majority remain stable and harmless. However, about 1 in 9000 naevi can transform into melanoma per year in a white population, and the diagnostic challenge for clinicians is to identify that transformation as early as possible [7]. Classic risk factors for transformation are summarised by the well-known TFSOM-DIM mnemonic [8]—“To Find Small Ocular Melanoma-Doing IMaging”:

- Thickness over 2 mm
- Fluid (subretinal)
- Symptoms
- Orange pigment
- Melanoma hollow (on ultrasound)
- DIaMeter > 5 mm (on fundus photography)

Accurate timely diagnosis is critical, as prompt therapeutic intervention can substantially reduce disease-related mortality [9]. Compelling evidence from research conducted in Denmark and Liverpool suggests that the treatment of smaller tumours may reduce the risk of metastatic disease and mortality attributable to the cancer [10,11]. However, differentiating an early choroidal melanoma from a benign nevus is often subtle and subjective. Ocular oncologists rely on multimodal imaging, fundus photography, B-scan ultrasonography, optical coherence tomography (OCT), and fluorescein angiography (FA) to assess the risk of such lesions.

Artificial intelligence is emerging as a tool that may support clinicians in this nuanced decision-making. In the past decade, AI has transformed screening and triage for diabetic retinopathy [12], retinopathy of prematurity (ROP) [13], and glaucoma detection [14]. We look to assess its usage and efficacy in the detection, screening, and monitoring of choroidal melanomas and how its implementation may facilitate more remote tele-medicine assessments of choroidal lesions.

Our review focuses exclusively on choroidal melanoma, the most prevalent subtype of uveal melanoma, which exhibits distinct clinical, imaging, and prognostic characteristics when compared with iris or ciliary body (CB) melanomas [15]. Previous reviews of AI in ocular oncology have typically catalogued algorithmic performance or considered uveal melanoma broadly. This review is distinct in providing a critical comparative synthesis that highlights methodological limitations, dataset diversity, algorithmic challenges, and translational barriers specific to choroidal melanoma. We also propose a conceptual framework and outline forward-looking research priorities to move the field from promise to practice.

## 2. Overview of Artificial Intelligence in Ophthalmology

Artificial intelligence encompasses a broad range of computational methods, with machine learning (ML) referring to algorithms that learn statistical patterns from data. Within ML, one important class of models is artificial neural networks, and deep learning (DL) denotes neural networks with many layers capable of learning hierarchical and highly complex representations. Traditional ML models—such as logistic regression, random forests, and XGBoost—typically use engineered input features, whereas DL models, particularly convolutional neural networks (CNNs), can automatically learn features directly from imaging data and have demonstrated strong performance in ophthalmic image analysis.

AI has rapidly advanced the delivery of healthcare, particularly in ophthalmology. As far back as 1976, a paper analysed a ML-based model, the causal-associational network (CASNET), for use in glaucoma consultations providing information on diagnosis and therapy [16]. More recently, IDx-DR, an AI system for diabetic retinopathy, became the first autonomous AI diagnostic device approved by the U.S. FDA [17]. In ROP, the i-ROP DL system has the ability to grade ROP and distinguish features such as plus disease with results comparable or better than expert diagnosis [18]. Across the board, it is the abundance of standardised image data, usually in the form of fundus photos or OCT scans, that makes this possible.

For choroidal melanoma, AI’s application is less straightforward. As it is a rare condition, there are significantly fewer diagnostic images for the dataset. Moreover, it can be difficult to distinguish some lesions between benign and malignant, and the diagnosis relies on features that are not always visible in a single image, such as tumour thickness, shape, growth rate, and internal reflectivity.

## 3. Studies Assessing AI in the Detection of Choroidal Melanoma

A structured comparative review was undertaken of peer-reviewed studies analysing the efficacy of various AI models in the classification of choroidal lesions and predictive risk of malignant transformation. These are outlined in Table 1. Commonly used metrics to assess the performance of ML and DL models include sensitivity, specificity, the F-1 score (measure of a model’s accuracy that balances precision and recall), and the area under the curve (AUC) (plots sensitivity against 1-specificity). The performance of the studies is highlighted in Table 2.

The number of images and patients described in each study reflects their training dataset and not the external validation dataset, where applicable.

Across all included studies, melanoma was designated as the positive class; therefore, the sensitivity, F1-score, and AUC refer to the correct identification of melanoma, while specificity reflects the correct identification of naevi. The data represents the performance metrics derived from the datasets used in each study’s reported training protocol.

We performed a structured comparative synthesis to evaluate robustness and translational readiness across published AI studies on choroidal melanoma. Each study was assessed across four domains: dataset diversity, ground truth definition, imaging modalities, and performance and validation.

### 3.1. Dataset Diversity

Dataset diversity is a key determinant of an AI model’s generalisability and clinical applicability. Models trained on small, homogeneous or single-centre datasets often exhibit high internal accuracy and have a susceptibility to overfitting but fail to perform consistently across different populations or imaging devices. Across the six reviewed studies, dataset design varied considerably. Zabor (2022), Tailor (2025), and Sabazade (2025) are notable for being the only studies that were multicentre [19,20,23]. Jackson’s model is significant as it addressed the issue of small datasets in the disease by looking at over 25,000 images, including healthy controls (1192 images), naevi (8671), and choroidal melanomas (18,510). Class balance, which is the relative proportion of melanomas to nevi, is crucial to avoid bias in model learning [25]. Marked class imbalances, such as the predominance of melanoma images in Jackson et al., can affect how a model learns if not explicitly addressed during training. Techniques such as stratified sampling or class weighting are therefore important to ensure balanced performance across classes.

None of the studies analysed included disease mimickers, such as choroidal haemangiomas, congenital hypertrophy of the retinal pigment epithelium (CHRPE), or peripheral exudative hemorrhagic chorioretinopathy (PEHC). As a result, their performance in differentiating these lesions from melanoma remains unknown, which may reduce specificity when applied in real-world diagnostic pathways. Wu et al. (2025) utilised a multimodal AI model which combined fundus photos, B-scan ultrasound, and radiology data to distinguish choroidal melanoma from mimickers like hemangiomas and metastases [26]. This study was beyond the scope of our review as it did not look at choroidal naevi and assess therisk of malignant disease; however, it is an interesting paper to mention as it used heatmaps to show which features drove the prediction. This is also known as explainable AI, and its transparency bridges the gap between AI’s statistical power and the ophthalmologist’s need to understand why a decision is made.

Age and sex were recorded in all studies, with the exception of Hoffman et al. (2024) [22]; However, the only studies to provide demographic distribution in terms of ethnicity were Dadzie (2024) and Tailor (2025) [20,21]. The cohort in Tailor et al. was predominantly white (94.6%), consistent with the established higher incidence of choroidal melanoma in this demographic. However, in Dadzie et al., white patients accounted for 64% of naevi and 84% of melanoma cases. The inclusion of racially diverse populations is critical for AI development, as variations in fundus pigmentation across ethnicities can significantly influence image characteristics and model performance [27].

### 3.2. Ground Truth Definition/Diagnosis

In AI model development, the reference standard (or ground truth) represents the true diagnosis against which model predictions are compared. Establishing a reliable reference standard is critical for valid training and evaluation yet remains challenging in ocular oncology, where histopathological confirmation is uncommon.

In the reviewed studies, the ground truth was derived through various methods, including expert clinical assessment, longitudinal confirmation of growth or stability, and, less frequently, histopathology. However, none of the studies reported standardised inter-grader agreement metrics, which may contribute to variability in feature importance and model performance across studies.

Consensus opinion by two ocular oncologists was utilised by Hoffmann et al. (2024) and Tailor et al. (2025) [20,22], while most studies described the use of multimodal imaging to support diagnosis. Four studies (Hoffmann, Sabazade, Tailor, and Zabor) incorporated longitudinal follow-up to assess lesion behaviour, with Tailor et al. specifically defining transformation as growth >0.5 mm within 24 months. Rarely, histopathologic confirmation was required.

### 3.3. Imaging Modalities

The imaging modalities used to train and validate AI models are critical determinants of diagnostic performance, influencing the features available for classification and the clinical relevance of model outputs. In ocular oncology, imaging plays a central role in differentiating benign from malignant lesions by revealing structural, vascular, and metabolic characteristics. Across the reviewed studies, variation exists in modality selection, ranging from single-modality approaches relying solely on colour fundus photography to comprehensive multimodal frameworks.

Single-modality studies, including Dadzie et al. (2024), Sabazade et al. (2025), and Hoffmann et al. (2024) [21,22,23], employed colour fundus photography, either in standard or ultra-widefield (UWF) formats, to provide surface-level detail of pigmentation, margins, and lesion extent. While this approach captures key morphological features and offers scalability for teleophthalmology, it lacks depth information and may overlook internal characteristics such as lesion thickness or subretinal fluid. Dadzie et al. used the DenseNet121 architecture on ultra-widefield (UWF) images to try to enhance DL accuracy through colour fusion strategies [21]. Colour fusion in deep learning combines multispectral inputs into a single colour image for easier analysis and interpretation [28]. Fusion can be performed across any set of imaging channels, including modalities such as infrared; however, in Dadzie et al. (2024) [21], colour fusion was specifically applied to the red, green, and blue channels exported from ultra-widefield Optos images. There are three main strategies used in image fusion in DL: early fusion (channels combined at the input stage), intermediate fusion (channels processed separately before merging), and late or decision-level fusion (outputs of each channel combined at the final layer).

Tailor et al. (2025) and Jackson et al. (2025) also analysed fundus autofluorescence (FAF) [20,24]. FAF highlights areas of lipofuscin accumulation, a biomarker associated with malignant transformation, and complements surface morphology with metabolic cues.

In contrast, multimodal approaches were adopted by the ML models of Tailor et al. (2025) and Zabor et al. (2022) [19,20]. They incorporated OCT and B-scan ultrasonography alongside fundus photography. These modalities provide additional information on lesion elevation, thickness, internal reflectivity, and associated retinal changes such as subretinal fluid, parameters known to be integral to clinical risk stratification. Tailor et al. (2025) [20] developed the simple AI Nevus transformation system (SAINTS) model, which utilised XGBoost, a ML algorithm based on the gradient boosting framework that both builds and refines an ensemble of weak prediction models in a tree-based algorithm. It reaffirmed known risk features but also quantified risk precisely, helping flag which patients may need closer follow-up or early treatment. The top five features most predictive of conversion from naevus to melanoma were tumour thickness (1.1–1.48 mm), largest tumour basal diameter (4.74–6.49 mm), tumour shape (specifically dome-shaped), distance to optic nerve (5.17–5.94 mm), and subretinal fluid (SRF) extent (greater SRF can be a sign of early transformation) [20,29]. It is worth noting that three of these features required multimodal imaging. Zabor et al. (2022) developed a Lasso logistic regression model to predict the risk of malignancy of choroidal naevi based on features extracted from FP and US [19]. It emphasised classic risk factors—SRF, tumour height, proximity to the optic disc, and orange pigment—as the most significant in increasing the odds of malignancy. Again, B-scan ultrasonography and OCT were required for the assessment of tumour height and SRF, respectively.

### 3.4. Performance and Validation

The AUCs reported across the studies varied from 0.86 to 0.99, with the highest reported by Hoffman et al. (2024) [22], specifically their ResNet50 model. DL models outperformed ML models across the studies. Jackson et al. (2025) and Sabazade et al. (2025), both presenting DL models, showed that CNNs trained on large image sets can match or outperform clinicians’ diagnostic accuracy [23,24]. Jackson et al. (2025), which drew from the largest sample size, reported an AUC of 0.90 in distinguishing naevi from melanoma with an accuracy of 0.83 [24]. However, their model, which utilised RETFound, has not yet been externally validated and does not encompass an analysis of OCT or B-scan US. Sabazade’s model achieved an AUC of 0.89 with 100% sensitivity, as was demonstrated in some tests [23]. Sabazade et al. (2025) employed a custom U-net model, which was notable for being a multicentre and externally validated study, thereby reducing the potential for bias [23].

Dadzie et al. (2024)’s colour fusion model, which found intermediate colour fusion, yielded the best diagnostic accuracy with a sensitivity of 81% and specificity of 98% [21]. This was because by allowing separate extraction of features from individual colour channels prior to merging, each channel was able to contribute its own unique features. In contrast, early fusion combines channels at the input stage, which can dilute modality-specific features, whereas late fusion merges outputs only at the decision layer, missing opportunities for shared feature learning across channels. This study demonstrated the feasibility of employing DL for the auto-classification of UM and choroidal naevi, facilitating rapid diagnosis and triage of referrals. Such a capability may be particularly useful in primary or secondary units that lack on-site ocular oncology expertise, where timely identification of suspicious lesions can help prioritise appropriate referral pathways. However, this work was not externally validated, and its performance on real-world, heterogeneous datasets remains unknown.

Hoffmann et al. used the DL model ResNet50, where 762 colour fundus photographs captured on various fundus cameras were analysed to distinguish choroidal naevi from melanoma [22]. The best performance was achieved upon using images from a single imaging modality. They had excellent results with an accuracy of 90.9%, F1 score of 0.91, and an AUC of 0.99. This study was limited by a relatively small dataset and was not externally validated. Although the use of different fundus cameras introduced variability, incorporating both tri-channel red–green–blue imaging (e.g., Clarus) and bi-channel red–green imaging (e.g., Optos) better reflects real-world practice, where multiple device types are routinely used.

The two studies utilising ML models both were externally validated. Zabor et al. (2022)’s Lasso logistic regression model was developed from 123 patients and externally validated on 240 patients, achieved an AUC of 0.86 [19]. Tailor et al. (2025)’s top performing model, SAINTS, had an AUC of 0.86 (2356 patients) but achieved an AUC of 0.93 in their external validation cohort of 514 patients [20]. This demonstrates that the model’s performance is generalisable to independent data.

From a clinical standpoint, these performance metrics translate directly into patient-relevant outcomes. High sensitivity is necessary to avoid missed melanomas—where delayed diagnosis results in poorer outcomes, while high specificity reduces unnecessary referrals and imaging for benign naevi. AUC values above 0.90 indicate reliable discrimination across a range of decision thresholds, supporting their potential use in teleophthalmology triage and community-based screening.

## 4. Limitations and Challenges

Despite promising results, there are major barriers to routine AI use for choroidal melanoma. First, dataset limitations remain critical. Unlike diabetic retinopathy, where significantly more standardised images exist, uveal melanoma is rare [30]. Most AI models are trained on small, single-centre cohorts. This increases the risk of overfitting—models may learn site-specific quirks that do not generalise elsewhere. External validation is therefore crucial but often lacking. Notably, Tailor et al. (2025) and Zabor et al. (2022) did use independent datasets [19,20]. Still, true generalisability demands large, diverse, prospectively collected data.

Black-box algorithms pose another challenge. While CNNs can detect subtle imaging patterns, they do not always explain their decisions in ways that clinicians or patients can easily understand. This can hinder trust and uptake. Explainable AI methods such as attention maps and concept bottlenecks and are a key factor in Wu et al.’s (2025) paper [26].

In light of high diagnostic accuracy, AI could help frontline providers, such as community optometrists, triage suspicious lesions more effectively. Many patients with intermediate-risk naevi undergo lifelong monitoring. AI could make this more precise and streamline pathways into specialist centres. The SAINTS model outputs individualised risk estimates for progression, potentially informing personalised follow-up intervals [20]. A study funded by the NIH, Iddir et al., showed that ML could detect changes in tumour thickness or subretinal fluid on serial scans with AUCs near 0.98—demonstrating capacity to flag suspicious growth earlier than manual measurement alone [31].

However, AI will not replace clinical judgement and requires integration with human expertise to ensure accurate interpretation and appropriate patient care. Real-world images also vary in quality compared with those in retrospective studies; artefacts such as blur, misalignment, or poor illumination can reduce model performance. Most datasets come from single centres, so there is a risk that the model is learning site-specific artefacts rather than generalisable features. Responsibly deployed AI should communicate uncertainty and support clinician judgement. This issue is central to the rapidly evolving regulatory landscape governing the real-world medical use of AI. Both the European Union’s AI Act and the U.S. FDA’s proposed AI/ML SaMD (Software as a Medical Device) framework stress continuous monitoring, algorithm retraining, and explainability for high-risk medical applications [32,33]. These requirements reflect the need for AI to remain transparent and auditable. However, despite this, the area of medico-legal liability remains ill-defined and clear professional guidelines around accountability are necessary before AI tools can be integrated into routine ocular oncology practice. Additionally, ethical considerations, including the preservation of patient autonomy and ensuring informed consent regarding the use of AI in their diagnostic work-up, must also be addressed.

Practical barriers to AI’s roll-out, particularly to include community-based screening for choroidal naevi, include cost, hardware, software integration, and training. Many clinics, especially in low-resource regions, lack high-quality fundus cameras or ultrasound equipment. Teleophthalmology can help bridge this gap, but AI tools must be robust enough to handle lower-quality images and variable technician skill. In diabetic retinopathy screening, AI works because fundus photography is straightforward and consistent. For choroidal melanoma, however, B-scan ultrasound is often needed, which requires specialist skill.

## 5. Future Directions

To close the gap between proof-of-concept and daily practice, we will require coordinated multicentre data generation, transparent model design, and prospective validation. Federated learning is a key element for choroidal melanoma detection. By enabling training across multicentre datasets without transferring or sharing the original raw patient images, federated approaches allow models to learn from large and diverse cohorts while maintaining data privacy. This strategy helps overcome the small-sample-size limitation that affects rare diseases such as uveal melanoma. Platform-level solutions such as NVIDIA FLARE and Google’s TensorFlow Federated provide ready-made infrastructures for secure aggregation, making them suitable starting points for multicentre uveal melanoma collaborations.

Explainable AI will be essential for clinical adoption because regulatory frameworks increasingly mandate interpretability for high-risk diagnostic applications. Models must provide clinically meaningful explanations—such as heatmaps or concept-level outputs—that align with known risk features and support clinician accountability. Human-in-the-loop oversight will be required to ensure accountability and maintain standards. Prospective real-world validation is essential and large multicentre trials will be required to test models’ performances across diverse populations with different hardware and imaging platforms. Successful large-scale real-world validation has been demonstrated in ophthalmic AI systems such as Google’s Automated Retinal Disease Assessment (ARDA), which underwent multicentre clinical trials across diverse populations and clinical environments [34].

Future systems must be multimodal by design. Because melanoma risk stratification relies on tumour elevation, internal reflectivity, and lipofuscin activity, AI tools will require the integrated analysis of fundus photography, OCT, and B-scan ultrasound rather than single-modality pipelines. Additionally, risk stratification will have to be a dynamic process. Longitudinal AI tracking must monitor lesion evolution across these modalities and predict conversion timelines.

Stratifying confirmed choroidal melanomas that are at a heightened risk of systemic dissemination also remains a diagnostic challenge. The absence of BRCA-1-associated protein 1 (BAP1) expression and monosomy 3 in uveal melanoma are known to be associated with metastatic progression and poor survival [35]. A potential future application of AI in uveal melanoma diagnosis is the identification of choroidal melanomas at elevated risk of metastasis without reliance on immunohistochemical analysis of biopsy samples. Integrating imaging and genomic data during model training offers a transformative opportunity to develop AI systems capable of inferring genomic risk profiles from imaging biomarkers, potentially diminishing the need for invasive tissue sampling. Whilst the integration of imaging and genomic data into AI models remains unrealised, Luo et al. were able to develop a random forest model using longitudinal B-scan ultrasonography and clinical parameters in 454 uveal melanoma patients treated with brachytherapy, to predict 4-year metastasis risk and mortality [36]. With only a single follow-up record, the model achieved modest performance (AUC ~0.708 for mortality prediction). However, with additional follow-up data included, performance improved, reaching an AUC of 0.883 for mortality prediction and an AUC of 0.846 for metastasis prediction. Their findings demonstrate that incorporating serial imaging data markedly improves prognostic accuracy.

Multiparametric Magnetic Resonance Imaging (mp-MRI) has also been explored as a non-invasive modality to infer high-risk genomic and prognostic features in uveal melanoma [37,38]. Wei et al. analysed dynamic contrast-enhanced magnetic resonance imaging to differentiate tumour characteristics of metastatic and non-metastatic choroidal melanoma and found the K parameter, the transfer constant from the blood plasma to the extracellular space, to be significantly lower in those with metastatic disease. Similarly, Kamrava et al. imaged 16 patients with mp-MRI (diffusion-weighted and dynamic contrast–enhanced perfusion sequences) and found that tumours with >33% monosomy 3 exhibited significantly higher K^trans^ and v_e_ perfusion parameters compared with disomic tumours, suggesting that increased permeability is associated with chromosome 3 loss [37]. Thus, mp-MRI has potential as an in vivo imaging technique to assist in the prediction of those patients at risk of metastatic disease, and this could be incorporated into future AI-driven multimodal assessment frameworks for choroidal lesions.

AI is positioned to play a real-world role in the detection of choroidal melanomas, with the potential to transform care delivery and expand teleophthalmology. Implementation should follow a hub-and-spoke teleophthalmology model, in which community optometry sites capture standardised widefield fundus and OCT imaging, with centralised AI triage prior to ocular oncology review. This could help identify and prioritise suspicious naevi in rural or underserved regions, enabling earlier specialist referral for high-risk lesions, and has been successfully trialled in diabetic retinopathy [39]. Ongoing audit and outcome monitoring will be essential to ensure that such systems reduce unnecessary referrals while maintaining a zero-tolerance threshold for missed melanomas. Large-scale, prospective, real-world evaluation represents the next decisive step toward safe clinical deployment.

## 6. Conclusions

AI holds genuine promise for transforming how choroidal melanoma is detected and monitored. Modern risk models like SAINTS, robust Lasso regressions, and powerful CNNs have shown expert-level accuracy under ideal conditions. When integrated carefully, AI can standardise risk assessment, flag subtle changes, and extend specialist expertise to frontline providers. Significant hurdles remain, such as small datasets, limited external testing, practical deployment costs, and medico-legal uncertainty, all demanding careful navigation. However, with well-designed studies, robust validation, and careful integration into patient-centred workflows, AI will standardise risk assessment, accelerate the detection of early transformation, and expand specialist expertise to frontline providers.

## Figures and Tables

**Table 1:** Summary of key AI studies on the detection of choroidal melanoma.

Author	AI Type	AI Model	Imaging Modality	Number of Images (Patients)	Number of Centres
Zabor et al., 2022 [19]	ML	Lasso logistic regression	FP, US	NR (123). CN: 62 CM: 61	2
Tailor et al., 2025 [20]	ML	XGBoost (SAINTS), LGBM, Random Forest, Extra Tree	FP, FAF, SD-OCT, US	NR (2870) CN: 2870 CM: 128	2
Dadzie et al., 2024 [21]	DL	DenseNet121	UWF	798 (438) CN: 281 CM: 157	1
Hoffmann et al., 2024 [22]	DL	ConvNext Base, EfficientNet B4, ResNet50, Vision transformer (SAM weights)	FP, UWF	NR (762) CN: 340 CM: 422	1
Sabazade et al., 2025 [23]	DL	U-net	FP, UWF	802 (688) CN: 583 CM: 219	2
Jackson et al., 2025 [24]	DL	RETFound	FAF, UWF	27,181 (4255) H: 1192 CN: 8671 CM: 18,510	1

CM, choroidal melanoma; CN, choroidal naevi; DL, deep learning; FAF, fundus autofluorescence; FP, fundus photography; H, healthy controls; LASSO, least absolute shrinkage and selection operator; LGBM, light gradient boosting model; ML, machine learning; NR, not reported; OCT, optical coherence tomography; SAINTS, simple AI Nevus transformation system; US, ultrasound; UWF, ultra-wide field photography; and XGBoost, extreme gradient boosting.

**Table 2:** Comparison of performance metrics across the studies.

Author	Sensitivity	Specificity	F1-Score	AUC
Zabor et al., 2022 [19]	NR	NR	NR	0.85
Tailor et al., 2025 [20] (SAINTS; XGBoost)	0.87	0.98	0.54	0.86
Dadzie et al., 2024 [21]	0.81	0.98	0.85	0.95
Hoffmann et al., 2024 [22] (ResNet50)	0.90	0.91	0.91	0.99
Sabazade et al., 2025 [23]	1.00	0.74	0.77	0.89
Jackson et al., 2025 [24]	0.79	0.87	0.84	0.90

AUC, area under the curve; NR, not reported; SAINTS, simple AI Nevus transformation system; and XGBoost, extreme gradient boosting.

## Data Availability

No new data were created or analyzed in this study. Data sharing is not applicable to this article.
